# Exploring the Acceptability, Feasibility, and Effectiveness of a Digital Parenting Program to Improve Parental Well-being After the Christchurch Earthquakes: Cluster-Randomized Trial

**DOI:** 10.2196/37839

**Published:** 2023-04-27

**Authors:** Liesje Donkin, Sally Merry, Stephanie Moor, Anna Mowat, Sarah Hetrick, Sarah Hopkins, Kara Seers, Chris Frampton, Lucy D'Aeth

**Affiliations:** 1 Department of Psychological Medicine, School of Medicine, Faculty of Medical and Health Sciences Waipapa Taumata Rau, The University of Auckland Auckland New Zealand; 2 Auckland University of Technology Auckland New Zealand; 3 Department of Psychological Medicine University of Otago Christchurch New Zealand; 4 Real Parents Christchurch New Zealand; 5 A Better Start: E Tipu e Rea National Science Challenge Wellington New Zealand; 6 Centre for Youth Mental Health University of Melbourne Melbourne Australia; 7 The National Public Health Service (Te Mana Ora) Te Whatu Ora Christchurch New Zealand

**Keywords:** psychology, eHealth, app, parenting, digital health, children, parent, well-being, earthquake, cluster-randomized trial, distress, child, mental health

## Abstract

**Background:**

Up to 6 years after the 2011 Christchurch earthquakes, approximately one-third of parents in the Christchurch region reported difficulties managing the continuously high levels of distress their children were experiencing. In response, an app named Kākano was co-designed with parents to help them better support their children’s mental health.

**Objective:**

The objective of this study was to evaluate the acceptability, feasibility, and effectiveness of Kākano, a mobile parenting app to increase parental confidence in supporting children struggling with their mental health.

**Methods:**

A cluster-randomized delayed access controlled trial was carried out in the Christchurch region between July 2019 and January 2020. Parents were recruited through schools and block randomized to receive immediate or delayed access to Kākano. Participants were given access to the Kākano app for 4 weeks and encouraged to use it weekly. Web-based pre- and postintervention measurements were undertaken.

**Results:**

A total of 231 participants enrolled in the Kākano trial, with 205 (88.7%) participants completing baseline measures and being randomized (101 in the intervention group and 104 in the delayed access control group). Of these, 41 (20%) provided full outcome data, of which 19 (18.2%) were for delayed access and 21 (20.8%) were for the immediate Kākano intervention. Among those retained in the trial, there was a significant difference in the mean change between groups favoring Kākano in the brief parenting assessment (*F*_1,39_=7, *P=*.012) but not in the Short Warwick-Edinburgh Mental Well-being Scale (*F*_1,39_=2.9, *P=*.099), parenting self-efficacy (*F*_1,39_=0.1, *P=*.805), family cohesion (*F*_1,39_=0.4, *P=*.538), or parenting sense of confidence (*F*_1,40_=0.6, *P=*.457). Waitlisted participants who completed the app after the waitlist period showed similar trends for the outcome measures with significant changes in the brief assessment of parenting and the Short Warwick-Edinburgh Mental Well-being Scale. No relationship between the level of app usage and outcome was found. Although the app was designed with parents, the low rate of completion of the trial was disappointing.

**Conclusions:**

Kākano is an app co-designed with parents to help manage their children’s mental health. There was a high rate of attrition, as is often seen in digital health interventions. However, for those who did complete the intervention, there was some indication of improved parental well-being and self-assessed parenting. Preliminary indications from this trial show that Kākano has promising acceptability, feasibility, and effectiveness, but further investigation is warranted.

**Trial Registration:**

Australia New Zealand Clinical Trials Registry ACTRN12619001040156; https://www.anzctr.org.au/Trial/Registration/TrialReview.aspx?id=377824&isReview=true

## Introduction

### Background

Following the 2011 Christchurch earthquakes, approximately one-third of parents continued to report elevated distress in their children up to 6 years later [1]. Unfortunately, the higher rates of distress after the earthquakes placed increased demand on the resources of a region that was, and still is, struggling to cope with postearthquake recovery. This resulted in many parents being unable to access services to support their children, thereby increasing parental distress and decreasing parenting confidence. Parents are central to helping their children learn to manage their emotions with parental self-efficacy, well-being, and confidence being linked to the well-being of the child [2], child adjustment, and child socioemotional development. Thus, improving parental confidence and well-being would also likely benefit the child.

Digital interventions can provide education and deliver interventions to facilitate parenting in an effective manner [3,4]. Examples of effective parenting interventions delivered by technology include those for reducing disruptive behaviors and for increasing parental self-efficacy [5]. Parents report that parenting apps help to provide reassurance [6], provide information about their own mental and physical health [5] and that of their child’s development [7], and are a means of getting support [8] in a manner that may reduce the barriers associated with traditional care [9].

Parents report a high level of satisfaction with digital interventions as a means of delivering information [10,11]. However, satisfaction does not necessarily lead to improved outcomes, and there is significant variability in the design process and theoretical underpinnings of the available digital interventions. Interventions with a sound theoretic base have shown to be more effective [12], and those that have been co-designed appear to be well received [9,13-15] and may better meet the needs of parents than those designed without consultation. Therefore, digital interventions that have a robust design process, a good theoretical underpinning, and are designed with the prospective audience in mind are more likely to be engaging and effective.

Although there were some parenting digital interventions available that could provide support, they were not developed for the population of New Zealand. In particular, interventions developed elsewhere did not consider Te Ao Māori (the Māori worldview) and would potentially be seen as less relevant and engaging for Māori, the indigenous population in New Zealand. This is particularly problematic, as the Māori face many disparities in health and well-being and need more engaging supports.

As a result of this, and with philanthropic funding, the Kākano parenting app was co-designed and developed with parents and local Māori advisors in the Christchurch region. The aim (after consultation with parents and other stakeholders) was to facilitate and increase parental confidence in managing children’s “big emotions” and supporting children through distress. An iterative design process using 4 focus groups of parents and community stakeholders was used to develop a prototype that was then tested in a small pilot trial. Initial data from the pilot testing of Kākano provided encouraging feedback about the usability and acceptability of Kākano. These results informed further development of the intervention, including shortening of intervention duration from 8 weeks to 4 weeks. The results of the development process will be reported elsewhere.

The final Kākano app drew on 3 key principles of Te Ao Māori to help families set weekly goals and facilitate family conversations about what values were important to them as a family. This study aimed to determine whether the Kākano app was (1) acceptable to parents that use it, (2) a feasible way to deliver parenting support, and (3) effective at improving parenting confidence and well-being.

### Final Kākano Intervention

The result of the co-design was a web-based app called Kākano (meaning seed in Māori). This name was chosen based on the focus group discussions and helped encapsulate the central theme of growth from the local iwi (tribe) representative. The iwi representative also discussed the name and underlying principles of the app with a group of local kaumātua (elders) to ensure that they were supportive of the concepts and naming of the app.

The final app drew on 3 key principles of Te Ao Māori (the Māori worldview) to help families set weekly goals and to facilitate conversations about what values were important to them as a family. The three Te Ao Māori principles used for goal setting were (1) Tika (doing things in the right way and order), (2) Pono (doing things with honesty and integrity), and (3) Aroha (doing things with love, compassion, sense of service to others, and joy). Tika, Pono, and Aroha are the key components of Mana (prestige/honor) within Te Ao Māori. The use of these 3 goals as a structure for the program was proposed by a group of local kaumātua (Māori leaders/elders) and welcomed by most of the parents surveyed, particularly those who were Māori.

Families were able to choose from a range of fun, strengths-based goals, which were in line with these values and consistent with the empirical evidence about supporting children who experience emotional and behavioral dysregulation. Families using the app were supported to enhance their family culture through intentionally working together on strengths they value and setting weekly goals to be “helpful,” “fun,” “adventurous,” “caring,” or “kind.”

There was also a “Coolers” section that offered a range of evidence-based and child-recommended resources for behavioral management to use when the family was experiencing problems with their children’s emotional and behavioral dysregulation. Information was presented in “bite-sized” pieces with skills suitable for rapid uptake and utilization. This approach was used because it was considered to fit the lives of busy parents and was in line with the latest technology approaches for information delivery [16].

Progress was marked at a weekly family meeting where the goals were reviewed and reset. These reviews and progress were visually demonstrated on the app by graphics depicting the weekly growth of the seed into a mature vine over 4 weeks.

The app also used common Māori words throughout. Māori participants’ feedback was that this made them feel that the app was developed specifically for them, while non-Māori participants did not particularly notice the use of Māori in the app (likely a reflection of the frequent use of Māori words in day-to-day language use in New Zealand).

**Figure 1:**
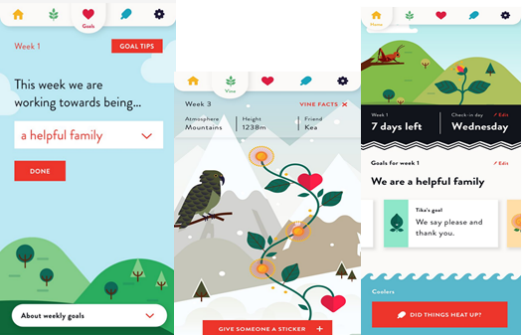
Screenshot of Kākano app.

Participants were given access to the site by a web link and then encouraged to use the app weekly for review and goal setting for a minimum of 4 weeks (the intended usage period of the app). Participants received weekly reminders to use the app. Figure 1 presents imagery from the app.

## Methods

### Overview

**Figure 2:**
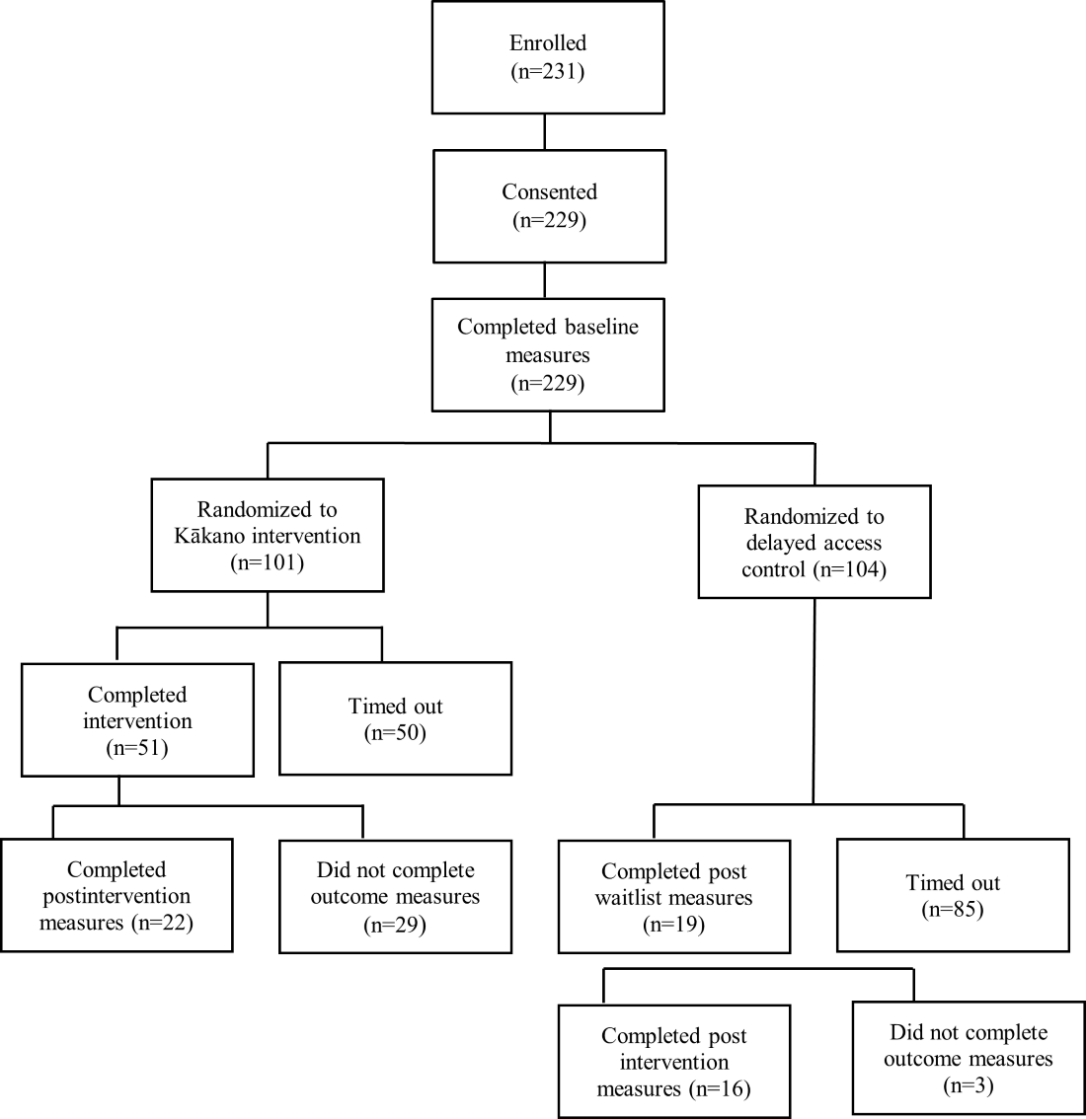
Participant flow through the Kākano trial.

The acceptability, feasibility, and effectiveness of Kākano were evaluated using a cluster-randomized delayed access controlled trial from July 2019 to January 2020 (Figure 2). Initially, it was envisaged that the study would be a stepped-wedge effectiveness trial consisting of 2 cohorts of data collection aiming to recruit 80 families each. However, it became obvious early in the recruitment that achieving near-simultaneous recruitment of a cluster of schools was not feasible. The first cohort took much longer than anticipated due to difficulties with engaging schools and local communities and obtaining consent. The design was therefore modified to a pre-post comparison of immediate access and delayed access groups, with the delayed access groups getting the intervention after the delay, primarily to get additional acceptability, feasibility, and effectiveness data.

Schools serving families representative of the target population for this app (a high proportion of Māori families or communities particularly affected by the earthquakes) were identified in the Christchurch region. The identified and consenting schools were block randomized to allow parents to receive either immediate or delayed access to Kākano.

After the delayed access period of 4 weeks, families randomized to receive the delayed intervention received an email or SMS text message (according to their preference) alerting them that the intervention was available after repeating the baseline measures.

Once families received access to the intervention, they were asked to complete postintervention measures at 4 weeks regardless of whether they completed any sessions of Kākano. To encourage completion of the measures, families who completed the data collection were entered into a draw to win a weekend family holiday valued at approximately NZ $400 (US $250).

Consent to participate, acceptance of the terms and conditions of the study, and completion of baseline measures were completed via the internet before accessing the app.

### Ethics Approval

This study was granted ethics approval from the Health and Disabilities Ethics Committee (approval 9/NTB/75) and was registered through the Australian New Zealand Clinical Trial Registry ACTRN12619001040156.

### Participants

The inclusion and exclusion criteria for this study are presented in Textbox 1.

**Inclusion and exclusion criteria for this study.**

**Inclusion criteria**

- Parents or caregivers of 1 child or more aged 5 to 12 years attending one of the identified recruitment schools
- Parents who have access to a web-enabled technology compatible with installing and running the app
- Parents who self-report struggling with parenting or wish to learn more skills to aid their parenting
- Parents aged 18 years and up

**Exclusion criteria**

- Parents receiving public or private parenting support or training currently or in the past 1 year
- Parents who are unable to read, write, or speak conversational English

### Measures

#### Demographic Information

Demographic information collected at baseline included the number of people living in the home, the number of children at home, the ages and ethnicity of the children at home, the age and ethnicity of the participating parent, the school decile (a New Zealand measure of the socioeconomic status of the population zoned for the school), and the name of the school of the oldest child aged 5 to 12 years.

#### Outcome Measures

Measures were completed before accessing Kākano at baseline (T0), after the primary intervention (T1), and 4 weeks later (postintervention T2) for the delayed access group.

Acceptability was assessed by measuring the participants’ satisfaction with the intervention. Parents were asked to complete a brief purpose-designed questionnaire to rate their satisfaction with the app intervention, including what they liked/did not like about the intervention, which aspects they found most/least helpful, whether they would recommend it to their friends, how it could be improved in the future, and whether any culturally specific issues needed to be addressed.

Feasibility was assessed using measures of compliance. We used computer-generated information on the number of components completed the timing of uptake and time spent on the resource.

We also measured the app’s effectiveness in improving parenting confidence and well-being.

There were 3 primary outcome measures of effectiveness: the Brief Assessment of Parenting Scale (BAPS), the Short Warwick-Edinburgh Mental Well-being Scale (SWEMWBS), and the Brief Parental Self-Efficacy Scale (BPSES). These scales are further detailed in the subsequent paragraphs.

BAPS is a purpose-designed scale created to assess parenting skills and confidence. It was developed based on the Parenting Sense of Competence (PSOC) scale [17] and the Weekly Assessment of Child Behavior-Positive (WACB-P) scale [18]. The scale was developed by a research team at the University of Auckland to reduce the time needed to complete assessments, and it consists of 10 items scored on a 5-point scale ranging from strongly agree to strongly disagree. All but 1 item are reversed scored and totaled to give a total score. Examples of items include “My relationship with my child(ren) is close and warm” and “I use positive language when my child(ren) are playing.”

SWEMWBS is a robust, widely used 7-item psychometric scale that focuses on the positive aspects of mental health [19]. It has shown to be largely free of bias and has been validated in numerous populations. The items are scored on a 5-point scale that is totaled to give a score between 7 and 35. The scale has been shown to have good internal consistency, convergent validity, and discriminant validity [20].

BPSES assesses a parent’s belief that they can effectively perform or manage tasks related to parenting their child. The scale is recommended by the Child Outcomes Research Consortium for use in the evaluation of parent training. The scale is not normalized and is intended as a change index within an individual.

The secondary outcome measures of effectiveness included the Family Adaptability and Cohesion Scale IV (FACES-IV) and the PSOC.

FACES-IV was developed to evaluate the adaptability and cohesion dimensions in family interactions [21]. The FACES-IV Package includes the Family Communication Scale and the Family Satisfaction Scale [22]. FACES-IV has been shown to have good reliability and validity [21]. The 9 FACES items used by the large Growing Up in New Zealand study [5] were used, as these have norms for the New Zealand population.

The PSOC consists of 17 parent-rated items on a range of parenting domains. Responses are on a 6-point scale ranging from “strongly disagree” to “strongly agree.” Scoring for some items is reversed, so for all items, higher scores indicate greater parenting self-esteem. The scale developers, Johnston and Mash [17], have reported internal consistencies of .75 for the Satisfaction scale and .76 for the Efficacy scale. The PSOC has been widely used across populations and ethnicities. In a recent systematic review of parenting measures, it was found to perform comparatively well across studies [23]. The PSOC was modified for this study from the assessment of parenting 1 child to parenting 1 or more children to fit in with the study aims. Table 1 shows the schedule of the data collection.

**Table 1:** Outcome measure data collection points for DA^a^ waitlist and IA^b^ to the Kākano intervention groups at the 3 data collection points^c^.

Measure			Baseline (T0)				Postintervention (T1)					Postintervention (T2)
			DA		IA	DA			IA		DA	
Demographic variables			✓		✓							
**Outcome measures**												
	BAPS^d^	✓		✓		✓		✓		✓		
	SWEMWBS^e^	✓		✓		✓		✓		✓		
	BPSES^f^	✓		✓		✓		✓		✓		
	FACES-IV^g^	✓		✓		✓		✓		✓		
	PSOC^h^	✓		✓		✓		✓		✓		
**Satisfaction measures**												
	Satisfaction with the intervention							✓		✓		
**Feasibility measures**												
	Number of goals set							✓		✓		
	Number of components of the intervention visited							✓		✓		

^a^DA: delayed access.

^b^IA: immediate access.

^c^A check mark (✓) indicates that the corresponding measure was collected at that time point.

^d^BAPS: Brief Assessment of Parenting Scale.

^e^SWEMWBS: Short Warwick-Edinburgh Mental Well-being Scale.

^f^BPSES: Brief Parental Self-Efficacy Scale.

^g^FACES-IV: Family Adaptability and Cohesion Scale IV.

^h^PSOC: Parenting Sense of Competence.

For all measures except the satisfaction measures, data for the immediate access group were collected via the internet at baseline (T0) and postintervention (T1; 4 weeks after the baseline). For the delayed access group, these were collected at baseline (T0) and repeated after the 4-week delayed access period (T1) and postintervention (T2; 4 weeks after the start of the intervention, 8 weeks after baseline).

The uptake and effectiveness trial was managed using a custom-made web-based portal [24], which was developed as part of the Health Advances Through Behavioral Intervention Technologies project funded jointly by the National Science Challenge, A Better Start: E Tipu e Rea, and Cure Kids.

### Randomization and Blinding

Randomization was computer-generated in advance by an independent member of the research team. Participants were block randomized based on the school that the eldest child (aged between 5 and 12 years) attended.

### Statistical Methods and Analysis

#### Power Calculation

Data from a pilot study feasibility study were used to estimate sample size, with original approximates estimates of about 240 (120 in each randomized group). This was based on an effect size of 0.25 to 0.30 using the estimated change in the BAPS score and changes in the PSOC score calculated from other universal parenting interventions.

#### Statistical Analysis

Statistical analysis was completed by author CF (a biostatistician), who oversaw the design and data analysis of the quantitative data. A linear mixed model was used to compare the changes before and after immediate access and delayed access between the study groups.

## Results

### Participants

In 2019, a total of 24 schools in the greater Christchurch area were invited to participate in the trial. Between July 10, 2019, and December 31, 2019, a total of 231 parents enrolled in the study, with 229 consenting to participate. Of these, 24 did not complete the baseline measures, resulting in 205 (88.7%) parents being randomized (101 to Kākano intervention and 104 to delayed-access control). A total of 41 (20 %) participants completed the preintervention and follow-up assessment—22 (21.8%) for Kākano and 19 (18.1%) at the end of the delayed access. Additionally, 164 (80%) timed out (ie, did not complete the postintervention questionnaires in the required time frame) (Figure 2). Because these participants timed out of the trial, there was no reason given for not completing the outcome measures.

Through the 205 parents who consented to participate in the trial and completed baseline measures, 453 children were enrolled by proxy, with each family having an average of 2.2 (SD 0.9) children and 4.1 (SD 1) people. Participants were predominantly female (n=185, 90.2%) and on average 38.9 (SD 9.1) years of age. Of the total sample, 83.4% (n=171) identified as New Zealand European, 7.3% (n=15) were Māori, and 1.5% (n=3) were Pasifika. The reported ethnicity of the eldest child in each family was 80.5% (n=165) New Zealand European, 7.3% (n=15) Māori, 1.4% (n=3) Pasifika, and 28.3% (n=58) other. Table 2 shows a breakdown of the participants’ demographics.

**Table 2:** Demographic characteristics of the study participants at baseline (T0).

Characteristics			Delayed access waitlist (n=104)		Kākano intervention (n=101)	Total (N=205)
Age (years), mean (SD)			37.1 (9.7)		40.6 (8.2)	38.9 (9.1)
Number of people in the house, mean (SD)			4 (1)		4.3 (1)	4.1 (1)
Number of children, mean (SD)			2.3 (0.8)		2.3 (0.8)	2.2 (0.9)
**Gender, n (%)**						
	Male	5 (4.8)		12 (11.9)		17 (8.3)
	Female	96 (92.3)		89 (88.1)		185 (90.2)
	Other	3 (2.9)		0 (0)		3 (1.5)
**Ethnicity of parent, n (%)**						
	Māori	12 (11.5)		3 (3)		15 (7.3)
	New Zealand European	82 (78.8)		89 (88.1)		171 (83.4)
	Pasifika	1 (1)		0 (0)		1 (0.5)
	Asian	1 (1)		2 (2)		3 (1.5)
	Other	8 (7.7)		7 (6.9)		15 (7.3)

### Acceptability

Numerous satisfaction questionnaires were included in the postintervention follow-up period, and these items were responded to by 22 participants. More than half (n=12, 54.5%) of the participants rated Kākano as helpful by rating it a score of 5/10 or higher on helpfulness, and 68.2% (n=15) of participants indicated that the digital delivery was helpful or extremely helpful. Goal setting was rated as the most helpful part of the app by over 90% of participants. Common barriers to the use of Kākano were time barriers (n=14, 63.6%) and family disengagement (n=2, 9.1%). One (5%) participant reported difficulty using the technology, and another (n=1, 5%) described finding the app confusing. Moreover, 77% (n=17) of participants that responded found the cultural fit of Kākano adequate by rating the app a 5/10 or higher for the item asking whether the cultural needs of the family were met by the app.

A total of 10 (45.45%) participants provided qualitative comments on Kākano in the postintervention period. The reported main reason for low use was forgetting to use the app and expecting the app to provide more reminders than it did. Two participants also noted difficulty with accessing the app due to not being able to use it offline or technical issues. Participants indicated that they would like more reminders to use the app, would like the app to be longer, and would like an option to pause use when they were not caregiving for their children (such as in a shared care arrangement).

### Feasibility Measures

There was no difficulty providing access to the app via the internet. A feasibility analysis based on usage of the app was completed using those that finished the trial (completers). Participants spent an average of 35.7 (SD 29.6) minutes using the app. There was a considerable range in participant usage, with some participants choosing to only briefly use the app (3 minutes) and others spending over an hour using the app (98 minutes) during the intervention period. The average number of sessions (discrete periods of using the app) completed by participants was 6.9 (SD 4.9; range 1-17) sessions. The number of activities and actions completed by participants in the app ranged from 4 to 78. Table 3 shows the usage metrics for participants that completed the trial.

When measures of usage were analyzed with regard to outcomes, no relationship between usage and outcomes was found.

**Table 3:** Usage metrics of Kākano for participants that completed the trial (n=22)^a^.

Usage metric	Mean (SD)	Median	Range
Time in the program (minutes)	35.7 (29.6)	25.5	3-98
Number of sessions	6.9 (4.9)	6	1-17
Average time per session (minutes)	5.6 (4.6)	4.5	0-18
Number of actions in the program	21.1 (16.3)	18.0	4-78

^a^Actions in the program were defined as changes or interactions with the app but do not include page views.

### Effectiveness Measures

There was a significant difference on the primary outcome variable of BAPS (*F*_1,40_=7, *P=*.012) but no significant difference by group on the SWEMWBS (*F*_1,40_=2.9, *P=.*099), BPSES (*F*_1,40_=0.1, *P=.*805), FACES-IV (*F*_1,40_=0.4, *P=.*538), or the PSOC (*F*_1,40_=0.6, *P=*.457) (Table 4).

A secondary analysis of the effectiveness of Kākano for those in the delayed-access control group showed a significant change for the BAPS (change score mean 1.9, SD 2.7; t_16_*=*2.8*, P*=.014) and the SWEMWBS (change score mean 1.5, SD 2.3; t_16_*=*2.6*, P=*.020) but not for the BPSES (change score mean 0.6, SD 1.4; t_16_*=*1.6*, P*=.132), FACES-IV (mean change score mean 0.8, SD 3.1; t_16_*=*1.1*, P*=.308), or PSOC (change score mean 3, SD 6.7; t_16_*=*1.8*, P*=.091).

**Table 4:** Mean and SD scores for the total sample with complete outcome data (n=41) for each outcome measure^a^.

Measure	Kākano intervention (n=22)				Delayed access waitlist (n=19)			Differences in change (intervention–waitlist)			Comparison of change	
	T0	T1	Change	T0		T1	Change	Mean (95% CI)	Effect size	*F* test (*df*)		Significance (*P* value)
BAPS^b^	38.3 (5.1)	40 (4.4)	1.7 (2.7)	38.4 (4.7)		37.9 (4.2)	–0.4 (2.4)	2.1 (0.5 to 3.8)	0.83	7 (1,40)		.01^c^
SWEMWBS^d^	24.5 (3.8)	25.6 (3.5)	1.2 (2.8)	23.9 (5)		23.6 (4.2)	–0.3 (2.8)	1.5 (–0.3 to 3.3)	0.53	2.9 (1,40)		.099
BPSES^e^	15.6 (2.2)	15.7 (2.9)	0 (2.3)	15.5 (2.6)		15.4 (2.1)	–0.1 (1.4)	0.2 (–1.1 to 1.4)	0.08	0.1 (1,40)		.81
FACES-IV^f^	28.9 (5)	29.5 (4.7)	0.6 (3.1)	29.6 (4.3)		28.5 (4.4)	–1.1 (2.6)	1.7 (–0.1 to 3.5)	0.58	0.4 (1,40)		.54
PSOC^g^	62.9 (10.4)	64.6 (7.8)	1.7 (6.5)	61.9 (13.6)		62.1 (12)	0.2 (6.5)	1.5 (–2.6 to 5.6)	0.24	0.6 (1,40)		.48

^a^Significant testing (*t* test) was conducted for change in scores from baseline (T0) to postintervention (T1) and for the difference between time points by groups (general linear model; *F* test).

^b^BAPS: Brief Assessment of Parenting Scale.

^c^Indicates analysis with significant results.

^d^SWEMWBS: Short Warwick-Edinburgh Mental Well-being Scale.

^e^BPSES: Brief Parental Self-Efficacy Scale.

^f^FACES-IV: Family Adaptability and Cohesion Scale IV.

^g^PSOC: Parenting Sense of Competence.

## Discussion

### Principal Findings

The results of this study were disappointing, with high levels of dropout. While Kākano was acceptable to those that completed the trial and delivery was feasible, few parents used it in its entirety. This may indicate that the delivery of parenting support may be acceptable and feasible, but that this app format may not engage everyone.

It was noted that half (n=5, 5%) of the participants used the intervention throughout the trial period, and even fewer (n=22, 22%) completed the outcome measures. It may be that some participants experienced benefits from using the app and thus continued to use it but chose not to complete the questionnaires, but we are not in a position to address this with the available data. Alternatively, these participants may not have found the app useful and therefore did not want to be involved in the trial. This could indicate low acceptability, feasibility, or effectiveness of the app. However, we are only able to speculate given these participants did not provide feedback on their reasons for not completing measures.

For those who did complete the outcome measures, there was some indication of improved parenting skill and confidence after 4 weeks of use and a possible trend toward significance in improved parental well-being. The remaining measures did not show significant changes, although the low number of participants completing the measures means we were underpowered to detect change. The results in the intervention group were consistent with the secondary analyses of the change in outcomes for the delayed access groups.

There were significant challenges in the recruitment and retention of families in this study. This is not uncommon in digital interventions, and despite our efforts to improve retention, it meant that the trial was underpowered. The emergence of the COVID-19 pandemic in New Zealand in early 2020, accompanied by strict lockdowns, meant that it was not feasible to extend the trial. A number of prospective participants did not complete the web-based registration process despite changes made to this process after the pilot, and it was clear that this was a major hurdle. Recruitment was done in the community by people outside of the research team, and this led to varied results, with some schools recruiting a large number of participants and others only recruiting a few. We noted a considerable variation in the methods of recruitment at schools, with some schools placing study advertisements in newsletters and web-based school platforms, some using word of mouth to recruit, and some using both methods. This variation in recruitment methods may have contributed to the difficulties with retention and meant that the clustering was not considered in the analysis. Finally, the Kākano app was fully automated and did not utilize any human support or reminders. This meant that parents were not actively followed up if they did not engage in the content, which likely impacted the engagement and persistence with the trial.

### Limitations

Despite using power calculations that allowed for attrition, the trial lost more participants than anticipated, and the size of this study was small. This makes it hard to determine the true effect of the intervention and limits the conclusions that we can draw about its effectiveness. The low numbers of participants that were Māori and Pasifika also mean we cannot determine whether Kākano is acceptable, feasible, or effective for Māori and Pasifika families.

It is also noted that the high attrition in this trial, although consistent with other mobile trials, means that results about acceptability need to be cautiously interpreted. Without participants giving reasons for not completing outcome measures or some form of qualitative analysis, it is difficult to determine if this attrition was due to the app or the nature of the trial.

### Comparison With Prior Work

To date, there are relatively few trials exploring apps for parenting confidence and well-being [3], but there is a growing field in the wider area of digital interventions for parenting [5,25]. This paper contributes to this field and highlights the difficulties of engaging parents in an ongoing way with digital interventions. Our data provide some tentative support for the idea that brief engagement with an app may be beneficial, and this is in line with other reports that microinterventions may be helpful for mental health [16] and particularly important for parents who face time constraints.

### Conclusions

This study aimed to assess Kākano, an app co-designed with parents to improve parenting confidence. We had significant difficulties retaining participants, despite the app being designed specifically with and for parents. Among the minority of parents who completed the trial, there was some evidence that Kākano improved parental well-being. Parents who used the app and provided feedback found it engaging and helpful. Given the low cost and ability to provide apps to a wide population, as well as the lack of harm likely to arise from an app of this kind, using this approach to support parental well-being is still worth considering.

## Appendix

Multimedia Appendix 1 CONSORT eHealth Checklist (V 1.6.1).

## Abbreviations

BAPS: Brief Assessment of Parenting Scale
BPSES: Brief Parental Self-Efficacy Scale
FACES-IV: Family Adaptability and Cohesion Scale IV
PSOC: Parenting Sense of Competence
SWEMWBS: Short Warwick-Edinburgh Mental Well-being Scale
WACB-P: Weekly Assessment of Child Behavior-Positive
